# Decrease in short‐latency afferent inhibition during corticomotor postexercise depression following repetitive finger movement

**DOI:** 10.1002/brb3.744

**Published:** 2017-06-09

**Authors:** Shota Miyaguchi, Sho Kojima, Ryoki Sasaki, Shinichi Kotan, Hikari Kirimoto, Hiroyuki Tamaki, Hideaki Onishi

**Affiliations:** ^1^ Institute for Human Movement and Medical Sciences Niigata University of Health and Welfare Niigata Japan

**Keywords:** motor evoked potential, postexercise depression, repetitive finger movement, short‐interval intracortical inhibition, short‐latency afferent inhibition, transcranial magnetic stimulation

## Abstract

**Introduction:**

This study aimed to clarify cortical circuit mechanisms contributing to corticomotor excitability during postexercise depression (PED) following repetitive nonfatiguing movement. We investigated changes in short‐latency afferent inhibition (SAI) and short‐interval intracortical inhibition (SICI) by paired‐pulse transcranial magnetic stimulation (TMS) during PED.

**Methods:**

A total of 16 healthy subjects performed repetitive abduction movements of the right index finger at 2.0 Hz for 6 min at 10% maximum voluntary contraction. We measured SAI evoked by pairing ulnar nerve stimulation with TMS (interstimulus interval, 22 ms) before and during PED (*n* = 10, experiment 1). We also measured SICI evoked by paired TMS (interstimulus interval, 2 ms) at 80% resting motor threshold (*n* = 10, experiment 2), and at 80% active motor threshold (*n* = 8, experiment 3) before and during PED.

**Results:**

Single motor evoked potential amplitude significantly decreased 1–2 min after the movement task in all experiments, indicating reliable PED induction. In experiment 1, SAI significantly decreased (disinhibited) 1–2 min during PED, whereas in experiments 2 and 3, SICI showed no significant change during PED.

**Conclusion:**

This study suggests that cholinergic inhibitory circuit activity decreases during PED following repetitive nonfatiguing movement, whereas GABA_A_ circuit activity remains stable.

## 1 INTRODUCTION

Corticomotor excitability changes after voluntary muscle contraction. The excitability of the primary motor cortex (M1) decreases after exhaustive exercise, causing muscle fatigue. This phenomenon has been defined as postexercise depression (PED) (Brasil‐Neto et al., 1993; Cunningham et al., 2016; Liepert, Kotterba, Tegenthoff, & Malin, 1996; Samii, Wassermann, Ikoma, Mercuri, & Hallett, 1996). PED is likely caused by intracortical mechanisms because spinal reflexes and peripheral and electrophysiological signs of subcortical activity, such as the *H* wave, *F* wave, *M* wave, and motor evoked potential (MEP) evoked by transcranial electrical stimulation do not change (Baumer, Munchau, Weiller, & Liepert, 2002; Brasil‐Neto et al., 1993; Samii et al., 1996). PED can be induced by repetitive movements without muscle fatigue (Avanzino et al., 2011; Bonato, Zanette, Fiaschi, & Rossini, 2002; Kluger, Palmer, Shattuck, & Triggs, 2012; Teo, Rodrigues, Mastaglia, & Thickbroom, 2012). In our previous studies, we found that PED was induced after voluntary repetitive finger movements paced at 0.5 Hz for 10 min and that PED also occurred after passive movement, suggesting the influence of proprioceptive feedback (Miyaguchi et al., 2013). Furthermore, we recently demonstrated that the degree of PED depended on muscle contraction level and type (Miyaguchi, Kojima, Kirimoto, Tamaki, & Onishi, 2016). In addition, Bonato et al. (2002) proposed that PED after nonfatiguing movements may reflect plastic neuronal reorganization produced by motor skill learning. However, the mechanisms of PED remain not completely clear, including whether inhibitory circuits in the cortex change during PED following nonfatiguing movements.

Several previous studies have examined the mechanism of PED using transcranial magnetic stimulation (TMS). In such studies, MEPs are recorded from the peripheral muscle when M1 is noninvasively stimulated by TMS, and MEP amplitude is used as an index of corticospinal pathway excitability. Moreover, paired TMS protocols can be used to evaluate inhibitory circuit function in the cortex. MEP amplitude decreases when a subthreshold conditioning stimulus is applied to the motor cortex a few milliseconds before a suprathreshold test stimulus, known as short‐interval intracortical inhibition (SICI). This inhibition depends on GABA_A_ receptor‐mediated cortical inhibition; therefore, SICI is used as an index of local GABA_A_ circuit activity in the cortex (Kujirai et al., 1993; Ziemann, Rothwell, & Ridding, 1996). Additionally, a TMS‐induced MEP is followed by a period of silence during muscle contraction, termed the cortical silent period (CSP), which is used as an index of GABA_B_ receptor‐mediated inhibition in the cortex and spine (Chen, Corwell, & Hallett, 1999; Ziemann, Netz, Szelenyi, & Homberg, 1993). A previous study using TMS reported PED induction after finger flexion‐extension movements for only 10 s associated with increased SICI (Teo et al., 2012). Moreover, CSP duration was reported to be extended during PED (Crupi et al., 2013), suggesting that PED is associated with increasing activity of cortical GABAergic circuits. However, the relationship between PED and excitability of cortical GABAergic circuits is unclear because SICI has also been reported to remain unchanged during PED (McDonnell & Ridding, 2006).

Short‐latency afferent inhibition (SAI), in which a preceding afferent stimulus transiently suppresses TMS‐induced motor output, is another index of inhibitory circuit excitability in the cortex. It is believed that SAI is caused by the inhibitory connections from the somatosensory cortex (S1) to primary motor cortex (M1) and is modulated by cholinergic inhibitory circuits in the cortex (Di Lazzaro, Pilato, Dileone, Tonali, & Ziemann, 2005b; Di Lazzaro et al., 2000, 2005a). Previous studies showed that the sensorimotor cortex activity decreased following a nonfatiguing repetitive finger task (Haavik Taylor & Murphy, 2007; Murphy, Haavik Taylor, Wilson, Oliphant, & Mathers, 2003) and that SAI is reduced by the decrease in excitability in the M1 and S1 (Kojima et al., 2015; Sasaki et al., 2016). Moreover, another study suggested that cholinergic circuits in the cortex are associated with the PED after a nonfatiguing repetitive motor task (Bonato et al., 2002). Based on these reports, we speculated that SAI decreased during PED following a nonfatiguing repetitive motor task. The purpose of this study was to examine changes in SAI and SICI during PED induced by repetitive nonfatiguing finger movement. This study suggests that distinct contributions of cortical GABAergic and cholinergic inhibitory circuits to motor cortex excitability changes following a nonfatiguing movement task.

## 2 MATERIALS AND METHODS

### 2.1 Experimental design

We conducted three experiments to assess changes in SAI and SICI during PED. In experiment 1, SAI was measured during PED, whereas in experiments 2 and 3, SICI was measured during PED using condition stimuli of 80% resting motor threshold (RMT) and 80% active motor threshold (AMT), respectively (referred to as SICI_80%RMT_ and SICI_80%AMT_).

### 2.2 Participants

Sixteen healthy subjects [11 males; age (mean ± standard deviation) 22.7 ± 2.5 years, range 20–30 years] participated in this study. None of the subjects were taking any medications or involved in upper limb sporting activities during the study. All subjects provided written informed consent. The study was approved by the ethics committee of Niigata University of Health and Welfare, and conducted in accordance with Declaration of Helsinki guidelines. During experiments, subjects were comfortably seated with the right shoulder in slight abduction, elbow in 90° flexion, and right forearm in pronation.

### 2.3 Transcranial magnetic stimulation and motor evoked potential recording

MEPs evoked by TMS before and after repetitive nonfatiguing finger movement were measured to evaluate corticospinal excitability. A Magstim 200 (Magstim Co, Dyfed, UK) was used as a magnetic stimulator, and a figure‐eight TMS coil (diameter, 9.5 cm) was placed tangentially at approximate 45° from the midline with the handle facing posterolaterally on each subject's skull. The optimal position for eliciting MEPs from the first dorsal interosseous (FDI) muscle was carefully determined by Visor 2 TMS Neuro‐navigation (EEMAGINE Medical Imaging Solutions GmbH, BER, DE), which can correctly identify the position of M1 by monitoring each subject's fMRI image. The RMT was defined as the lowest TMS intensity needed to elicit MEPs of 50 μV or more in at least 5 of 10 successive trials in the relaxed target muscle (Di Lazzaro et al., 2000). The AMT was defined as the stimulus intensity required to elicit MEP of 100 μV in at least 50% of trials during a slight isometric contraction (Di Lazzaro et al., 2000). In both cases, TMS was delivered at 0.20 Hz. The intensity of the stimulator output for the induction of MEP by single pulses (single MEP) was set at 110%, 115%, 120%, 125%, and 130%RMT. The right forearm was in pronation during MEP acquisition.

### 2.4 Electromyogram (EMG) recording

The surface EMG was recorded from the right FDI muscle using Ag/AgCl electrodes. The active electrode was positioned over the muscle belly and the reference electrode over the metacarpophalangeal joint. The ground electrode was wound around the right forearm. The signals were amplified (×100) by a preamplification system (A‐DL‐720‐140, 4 Assist, Tokyo, Japan), digitized at 10 kHz by an A/D converter (PowerLab 8/30, AD Instruments, Colorado, USA), filtered with a 15‐Hz high‐pass filter and stored on a personal computer for off‐line analysis using Lab Chart 7 (AD Instruments).

### 2.5 Experiment 1: Evaluation of short‐latency afferent inhibition (SAI)

A total of 10 subjects (age, 22.4 ± 1.6 years) participated in this experiment. Conditioning MEPs were measured in response to TMS delivered 22 ms after ulnar nerve stimulation (Teo, Terranova, Swayne, Greenwood, & Rothwell, 2009; Tokimura et al., 2000). We used an electrical stimulator (SEN‐8203, Nihon Kohden, Tokyo, Japan) and bar‐type stimulation electrode (length, 55 mm; width, 15 mm; electrode distance, 20 mm) to deliver square pulse stimuli through a bipolar stimulation probe fixed over the ulnar nerve at the wrist with the cathode positioned proximally. The stimulation electrode was firmly fixed by Velcro tape. Additionally, the position of the stimulus electrode was marked with a pen, and the fact that the electrode position did not shift before and after the motor task was confirmed. The stimulus was set at the lowest intensity required to produce an *M* wave (50 μV) at a pulse duration of 0.2 ms (11.0 ± 4.1 mA). The TMS intensity was set at 110%, 115%, 120%, 125%, and 130%RMT.

### 2.6 Experiment 2: Evaluation of the SICI_80%RMT_

A total of 10 subjects (age, 22.8 ± 1.6 years) participated in this experiment. Conditioned MEPs were induced by a TMS pulse delivered 2 ms after a conditioning pulse (Di Lazzaro et al., 2005a,b; Kujirai et al., 1993) at 80%RMT. The test stimulus intensities were set at 110%, 115%, 120%, 125%, and 130%RMT as in experiment 1.

### 2.7 Experiment 3: Evaluation of the SICI_80%AMT_

A total of eight subjects (age, 23.8 ± 2.9 years) participated in this experiment. Conditioned MEPs were measured using paired‐pulse TMS with conditioning stimulus intensity of 80%AMT. Test stimulation intensity and interstimulus interval were the same as in Experiment 2.

### 2.8 Repetitive nonfatiguing movement task

The motor task was described previously (Miyaguchi et al., 2016) and a schematic diagram is shown in Miyaguchi et al. (2013). The subjects performed isotonic repetitive abduction movements of the right index finger (from neutral to the end of abduction) for 6.0 min while maintaining the pace at 2.0 Hz using auditory feedback. The muscle contraction level was set at 10% of maximum voluntary contraction (MVC) by weight loading the index finger (Miyaguchi et al., 2013). The load was adjusted so the required contraction level was equal to the average amplitude of the smoothing EMG signals during the motor task. Prior to the movement task, subjects practiced repetitive abduction movements by watching EMG signals on a PC screen for approximately 10 s. Then, the subjects performed the motor task without watching EMG signals. Baseline MEP acquisition was started approximately 10 min after motor practice. We selected this 10‐min washout time based on previous findings that changes in cortical excitability after nonfatiguing repetitive movement return to baseline within 4–8 min (Miyaguchi et al., 2016; Teo et al., 2012).

### 2.9 Experimental Procedure

In each experiment, 12 single MEPs and 12 conditioned MEPs were randomly measured at five magnetic stimulation intensities of 110%–130%RMT before the motor task (pre). The total number of stimuli was 120 at pre. Then, the motor task was performed for 6 min. At 60–180 s (post 1–2 min) and 180–300 s (post 3–4 min) after the motor task, 12 single MEPs and 12 conditioned MEPs were again measured within each 2‐min epoch at a magnetic stimulation intensity of 130%RMT. Therefore, single MEPs and paired‐pulse MEPs were collected for each of the 12 waveforms at post 1–2 min and post 3–4 min. The total number of stimuli was 48 after motor task. The three experiments were performed with a break of at least 1 week.

### 2.10 Data analysis

Mean MEP amplitudes were calculated as peak‐to‐peak amplitudes of trials after excluding the largest and smallest. The MEP ratio (the conditioned MEP/single MEP ×100) was calculated to evaluate SAI and SICIs (SAI Ratio, SICI_80%RMT_ Ratio, and SICI_80%AMT_ Ratio). Both SAI and SICI have been reported to vary depending on MEP amplitude (Ni et al., 2011; Roshan, Paradiso, & Chen, 2003; Udupa, Ni, Gunraj, & Chen, 2009) and amplitude changes due to PED. Therefore, to compare SAI or SICI before and after the movement task independent of the influence of MEP amplitude, the single pre‐ and post‐MEP amplitudes must be adjusted to the same value by adjusting TMS intensity. However, it is difficult to adjust TMS intensity because PED only lasts 1–3 min after the motor task (Miyaguchi et al., 2016). Therefore, to measure the adjusted SAI and adjusted SICI, single MEPs and conditioned MEPs were measured at a TMS intensity of 110%–130%RMT before the task, and at 130%RMT at post 1–2 min and post 3–4 min. The value closest to the single MEPs at post 1–2 min and post 3–4 min (pre_adjust1_ and pre_adjust2_, respectively) was selected from among the single MEPs with 110%–130% RMT; the value of the conditioned MEPs obtained with the same intensity was used for the comparison of SAI or SICI before and after the motor task. In other words, the single MEPs were adjusted before and after the motor task using the single MEPs by 110%–130% RMT measured before the motor task. With this method, we compared SAI and SICI, which removed the influence of a single MEP size in this study.

The average amplitudes of the smoothing EMG signals for 60 s after the beginning of motor tasks (start_60 s) and for 60 s before the end of motor tasks (end_60 s) were normalized with the values obtained at MVC in each subject to confirm that muscle fatigue does not occur after performing motor tasks.

### 2.11 Statistical analysis

One‐way repeated measures analysis of variance (ANOVA) was used to compare single MEPs evoked by 130% RMT at pre, post 1–2 min, and post 3–4 min time points. Post‐hoc analysis was performed by Bonferroni correction. Single MEPs, each conditioned MEP, SAI Ratio, SICI_80%RMT_ Ratio, SICI_80%AMT_ Ratio, and TMS intensity before and after the motor task were compared by paired *t* test. The normalized EMG (start_60 s and end_60 s) were also compared by paired *t* test. Differences were considered statistically significant at *p *<* *.05 for all analyses. The effect sizes were indicated by Cohen's d.

## 3 RESULTS

### 3.1 Experiment 1: Changes in SAI during PED

**Figure 1:**
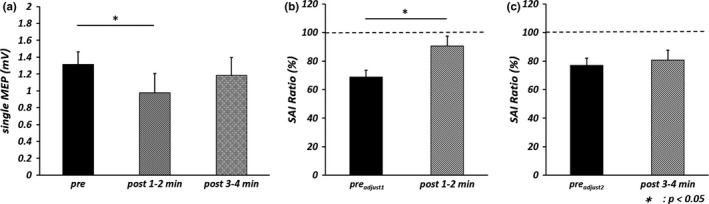
Mean single motor evoked potential (single MEP) amplitude and short‐latency afferent inhibition (SAI) Ratio before and after repetitive index finger abduction at 2.0 Hz for 2 min. The error bars indicate standard error. (a) Mean single MEP amplitudes evoked by transcranial magnetic stimulation intensity of 130% resting motor threshold (RMT) before the motor task (pre) and at two epochs after the task (post 1–2 min and post 3–4 min) in the experiment 1. Statistical comparisons by one‐way repeated measures analysis of variance (*p < .05). (b) SAI Ratio before and 1–2 min after the motor task. Statistical comparisons by paired *t* test (*p < .05). (c) SAI Ratio before and 3–4 min after the motor task. Statistical comparisons by paired *t* test

One‐way repeated measures ANOVA revealed a significant main effect of TIME [*F*_(2, 18)_  = 5.323, *p *<* *.05, partial η^2^ = .372] (Figure 1a), and post‐hoc analysis revealed that single MEPs evoked by 130% RMT at post 1–2 min were significantly smaller than that at pre (*p *<* *.05, *r* = .70). A paired *t* test showed that TMS intensity at post 1–2 min (65.8% ± 2.5%) (mean ± standard errors) was significantly larger than that at pre_adjust1_ (61.0% ± 2.4%) (*p *<* *.01, *r* = .88) and that TMS intensity at post 3–4 min (65.8% ± 2.5%) was significantly larger than that at pre_adjust2_ (61.5% ± 2.5%) (*p *<* *.01, *r *=* *.82). Table 1 shows the amplitudes of single MEPs and conditioned MEPs before and after the motor task. There was no significant difference in mean single MEP amplitude between pre_adjust1_ and post 1–2 min or between pre_adjust2_ and post 3–4 min by paired *t* test, indicating that single MEP amplitude was properly adjusted for comparison of SAI before and after the motor task. Moreover, a paired *t* test to compare single MEP and conditioned MEP amplitude at pre, post 1–2 min, and post 3–4 min (Table 1) revealed that conditioned MEPs were significantly smaller than single MEPs at pre_adjust1_ and pre_adjust2_ (pre_adjust1_: *p *<* *.01, *r *=* *.94; pre_adjust2_: *p *<* *.01, *r *=* *.76). However, no significant differences were found at post 1–2 min and post 3–4 min. Furthermore, SAI Ratio at post 1–2 min was significantly larger than that at pre_adjust1_ (*p *<* *.05, *r *=* *.62) (Figure 1b, c).

**Table 1:**
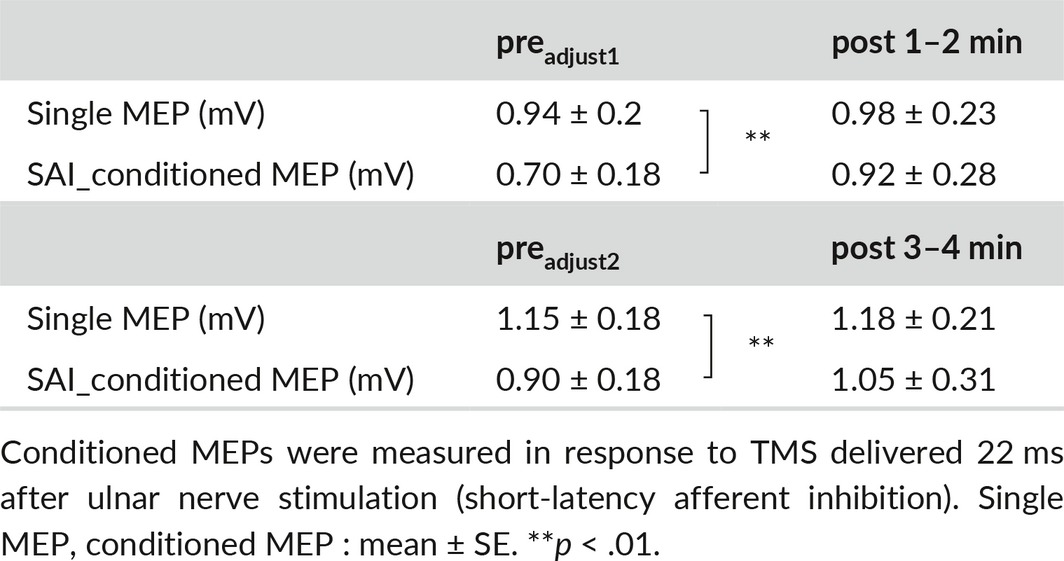
Single motor evoked potential (MEP) and conditioned MEP amplitudes before and after the movement task in experiment 1

### 3.2 Experiment 2: Change in SICI_80%RMT_ during PED period

**Figure 2:**
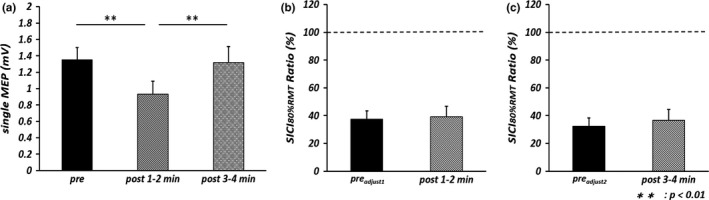
Mean single motor evoked potential (single MEP) amplitude and SICI_80%_ _RMT_ Ratio before and after repetitive index finger abduction at 2.0 Hz for 2 min. The error bars indicate standard error. (a) Mean single MEP amplitudes evoked by transcranial magnetic stimulation intensity of 130% resting motor threshold (RMT) before the motor task (pre) and at two epochs after the task (post 1–2 min and post 3–4 min) in the experiment 2. Statistical comparisons by one‐way repeated measures analysis of variance (**p < .01). (b) SICI_80%_ _RMT_ Ratio before and 1–2 min after the motor task. Statistical comparisons by paired *t* test. (c) SICI_80%_ _RMT_ Ratio before and 3–4 min after the motor task. Statistical comparisons by paired *t* test

One‐way repeated measures ANOVA revealed a significant main effect of TIME [*F*_(2, 18)_  = 8.750, *p *<* *.01, partial η^2^ = .493] (Figure 2a), and post‐hoc analysis demonstrated that single MEPs evoked by 130% RMT at post 1–2 min were significantly smaller than at pre (*p *<* *.01, *r* = .77) and post 3–4 min (*p *<* *.01, *r *=* *.77). TMS intensity at post 1–2 min (66.5% ± 2.8%) was significantly larger than that at pre_adjust1_ (61.8% ± 2.2%) (*p *<* *.01 by paired *t* test, *r *=* *.84), and TMS intensity at post 3–4 min (66.5% ± 2.8%) was significantly larger than that at pre_adjust2_ (63.8% ± 2.6%) (*p *<* *.01 by paired *t* test, *r *=* *.66). Table 2 shows the mean amplitudes of single MEPs and conditioned MEPs before and after the motor task. There was no significant difference in mean single MEP amplitude between pre_adjust1_ and post 1–2 min or between pre_adjust2_ and post 3–4 min, confirming that single MEP amplitude was properly adjusted for comparison of SICI_80%RMT_ before and after the motor task. Moreover, conditioned MEP amplitude was significantly smaller than single MEP amplitude at pre_adjust1_, pre _adjust2_, post 1–2 min and post 3–4 min by paired *t* test (pre_adjust1_: *p *<* *.01, *r *=* *.89; pre_adjust2_: *p *<* *.01, *r *=* *.93; post 1–2 min: *p *<* *.01, *r *=* *.92; post 3–4 min: *p *<* *.01, *r *=* *.95). However, there was no significant difference in SICI_80%RMT_ Ratio between pre_adjust1_ and post 1–2 min or between pre_adjust2_ and post 3–4 min (Figure 2b, c).

**Table 2:**
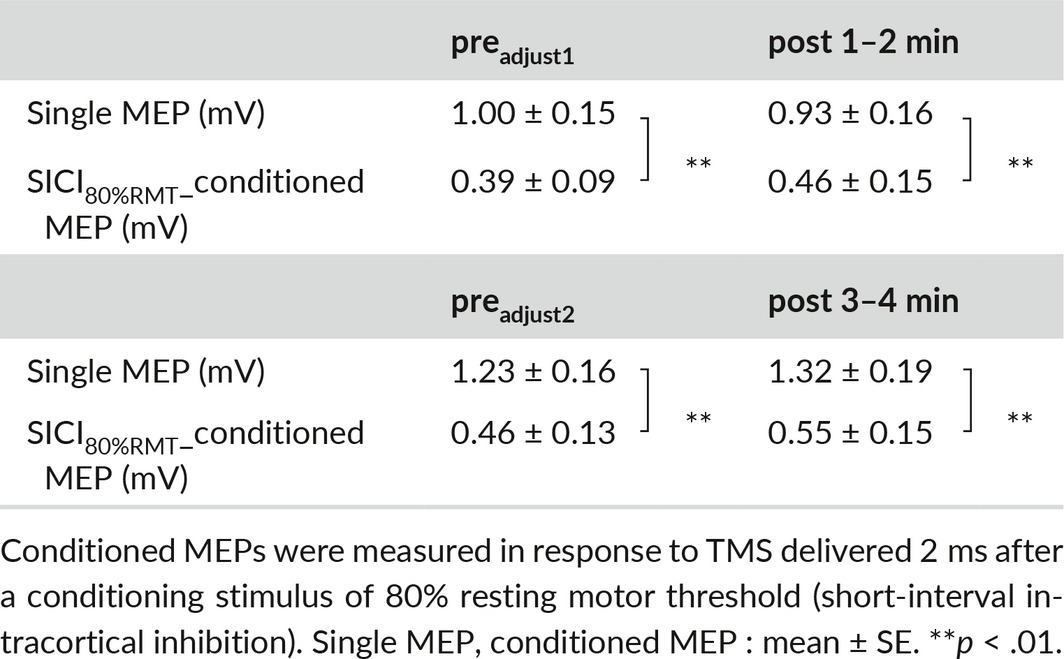
Single motor evoked potential (MEP) and conditioned MEP amplitudes before and after the movement task in experiment 2

### 3.3 Experiment 3: Change in SICI_80%AMT_ during PED period

**Figure 3:**
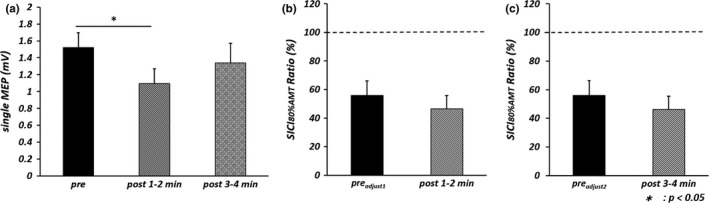
Mean single motor evoked potential (single MEP) amplitude and SICI_80%_ _AMT_ Ratio before and after repetitive index finger abduction at 2.0 Hz for 2 min. The error bars indicate standard error. (a) Mean single MEP amplitudes evoked by transcranial magnetic stimulation intensity of 130% resting motor threshold (RMT) before the motor task (pre) and at two epochs after the task (post 1–2 min and post 3–4 min) in the experiment 3. Statistical comparisons by one‐way repeated measures analysis of variance (*p < .05). (b) SICI_80%_ _AMT_ Ratio before and 1–2 min after the motor task. Statistical comparisons by paired *t* test. (c) SICI_80%_ _AMT_ Ratio before and 3–4 min after the motor task. Statistical comparisons by paired *t* test

One‐way repeated measures ANOVA revealed a significant main effect of TIME [*F*_(2, 15)_  = 4.177, *p *<* *.05, partial η^2^ = .358] (Figure 3a), while post‐hoc analysis demonstrated that mean single MEPs evoked by 130% RMT at post 1–2 min was significantly smaller than those at pre (*p *<* *.05, *r* = .70). There was no significant difference in TMS intensity between pre_adjust1_ (68.4% ± 2.6%) and post 1–2 min (71.0% ± 2.8%) or between pre_adjust2_ (69.5% ± 2.8%) and post 3–4 min (71.0% ± 2.8%) by paired *t* test. Table 3 shows the mean amplitudes of single MEPs and conditioned MEPs before and after the motor task. There was no significant difference in single MEP amplitude between pre_adjust1_ and post 1–2 min or between pre_adjust2_ and post 3–4 min, again confirming that single MEP amplitude was properly adjusted for comparison of SICI_80%AMT_ before and after the motor task. The conditioned MEP was significantly smaller than the single MEP at pre _adjust1_, pre _adjust2_, post 1–2 min, and post 3–4 min by paired *t* test (pre _adjust1_: *p *<* *.05, *r *=* *.75; pre _adjust2_: *p *<* *.01, *r *=* *.74; post 1–2 min: *p *<* *.01, *r *=* *.84; post 3–4 min: *p *<* *.05, *r *=* *.77). However, there was no significant difference in SICI_80%AMT_ Ratio between pre_adjust1_ and post 1–2 min or and between pre_adjust2_ and post 3–4 min (Figure 3b, c).

**Table 3:**
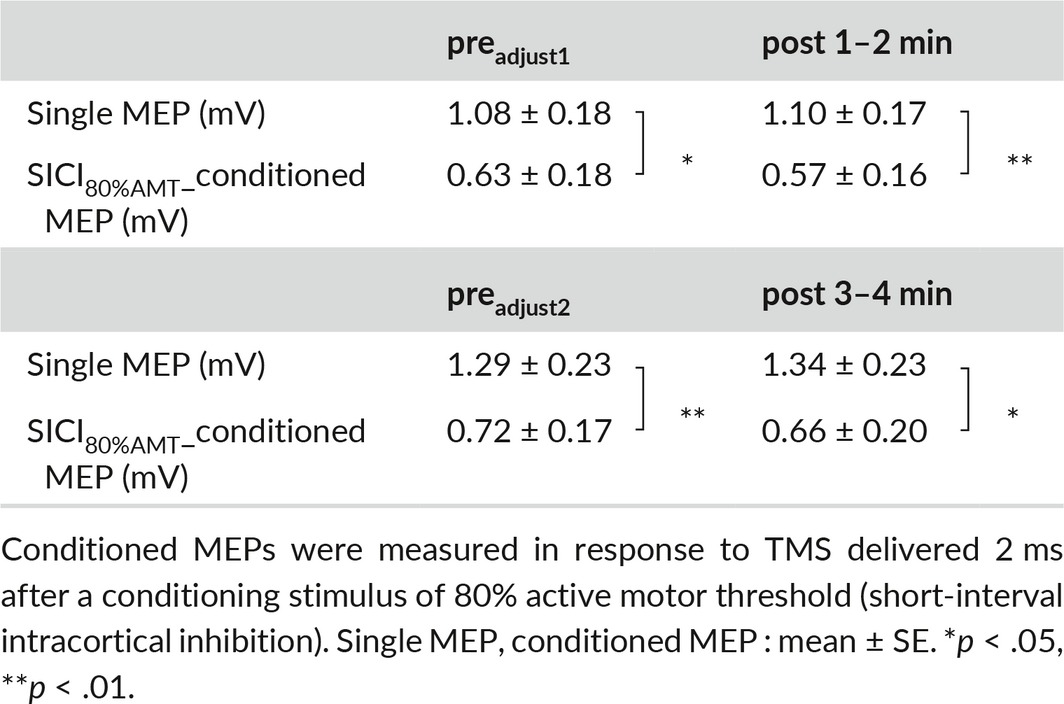
Single motor evoked potential (MEP) and conditioned MEP amplitudes before and after the movement task in experiment 3

### 3.4 EMG activity during motor tasks

Table 4 shows the normalized EMG activity in all experiments. A paired *t* test showed no statistical difference in the EMG activity at start_60 s and end_60 s in all experiments.

**Table 4:** Normalized electromyogram (EMG) activity at start_60 s and end_60 s in all experiments

	start_60 s	end_60 s
Experiment 1	8.3 ± 2.2	8.3 ± 2.2
Experiment 2	9.2 ± 3.0	8.7 ± 3.0
Experiment 3	7.6 ± 1.1	6.9 ± 1.5

Normalized EMG activity: mean ± SD (%).

## 4 DISCUSSION

We investigated changes in MEP amplitudes, SAI, and SICI after repetitive abduction movements of the right index finger to examine whether the inhibitory processes underlying SAI and SICI are altered during PED. Single MEP amplitudes decreased significantly for 1–2 min after the movement task, indicating transient PED. While SAI decreased significantly for 1–2 min after the movement task, SICIs did not change. Moreover, no significant difference was found in all experiments in EMG activity at start_60 s and end_60 s. Therefore, it is considered that muscle fatigue did not occur in all experiments. Although we have not performed pharmacological experiments, our findings suggest that the cholinergic inhibitory circuit activity may be transiently reduced during PED after nonfatigue movement, whereas the excitability of GABA_A_ circuits mediating SICI possibly remains stable during PED after nonfatiguing movement. The previous studies reported that PED following finger movement task at maximum frequency persists for 35 min (Zanette et al., 1995), and SICI decreases during PED following muscle fatigue task (Maruyama, Matsunaga, Tanaka, & Rothwell, 2006). The results of the present study differ from those of previous studies. Therefore, PED after nonfatigue movements may be different from that after a muscle fatigue task.

### 4.1 PED after repetitive nonfatiguing movement task

Single MEP amplitudes decreased significantly for 1–2 min after the movement task in this study. Many previous studies have reported that PED occurs after a brief repetitive nonfatiguing movement task (Avanzino et al., 2011; Crupi et al., 2013; Miyaguchi et al., 2013, 2016; Teo et al., 2012). Bonato et al. (2002) measured PED after thumb adduction‐abduction movement at 2.0 Hz for 1 min, and Teo et al. (2012) measured PED after index flexion‐extension for 10 s. Therefore, PED appears to be reliably induced by repetitive nonfatiguing movement lasting from seconds to minutes. The MEP amplitudes reflect excitability of the motor cortex, spinal cord, and periphery. Although previous studies have demonstrated that the excitability of spinal cord and periphery temporarily decreases after maximal voluntary contraction (Gandevia, Petersen, Butler, & Taylor, 1999; Khan, Giesebrecht, Gandevia, & Taylor, 2012; Petersen, Taylor, Butler, & Gandevia, 2003), it has not been reported that the excitability of the spinal cord and periphery change after nonfatiguing movement of low intensity (Avanzino et al., 2011; Teo et al., 2012). Therefore, it is considered that a decrease in the single MEP amplitudes after 10%MVC task in this study was caused by intracortical mechanisms, indicating transient PED.

### 4.2 The adjustment of TMS intensity

As SAI and SICI have been reported to vary depending on MEP amplitude (Ni et al., 2011; Roshan et al., 2003; Udupa et al., 2009), it is critical that single MEP amplitude is adjusted to the same value before and after the motor task by changing TMS intensity. No significant differences were found in single MEP amplitude between pre_adjust1_ and post 1–2 min and between pre_adjust2_ and post 3–4 min, indicating that the changes in SAI and stability of SICI after the motor task were not influenced by differences in MEP amplitude.

### 4.3 Change in SAI during PED period

The previous studies concluded that PED after nonfatiguing movement involves changes in GABAergic inhibitory circuit excitability because SICI increases and CSP is prolonged during PED (Crupi et al., 2013; Teo et al., 2012). However, our results showed that SAI decreased during PED following nonfatiguing finger movement task. Therefore, the present study is the first to reveal the change in cortical inhibitory circuit during PED after nonfatiguing movement. SAI is mediated by cholinergic inhibitory circuit excitability as evidence by loss of SAI under cortical cholinergic blockade (Di Lazzaro et al., 2000, 2005a,b). Therefore, our results suggest that cholinergic inhibitory circuit excitability is reduced during PED after nonfatiguing movement. Several previous studies have examined the relationship between SAI and M1 excitability. Although one study using TMS reported reduced SAI with decreasing M1 excitability (Sasaki et al., 2016), other studies using repetitive TMS found that SAI does not change during decreased M1 excitability (Fischer & Orth, 2011; Tsang et al., 2014). The results of the current study are consistent with those of the study by Sasaki et al. (2016) and suggest that PED is associated with reduced cholinergic inhibition. However, the relationship between reduced SAI and decreasing M1 excitability remains unclear. Tsang et al. (2014) reported that although continuous theta‐burst stimulation (cTBS) over M1 suppressed single MEP and did not alter SAI, cTBS over S1 decreased SAI. This previous study suggested that SAI is affected by S1 activity rather than M1. Therefore, the different mechanisms may be involved in the reduction in the SAI and M1 excitability following nonfatiguing movement. Several previous studies using electroencephalography have reported somatosensory evoked potential (SEP) changes after repetitive finger movement tasks (Andrew, Haavik, Dancey, Yielder, & Murphy, 2015; Haavik & Murphy, 2013; Murphy et al., 2003), suggesting alterations in primary somatosensory cortex (S1) excitability as well as M1 excitability. For instance, N30 peak amplitude, which reflects sensorimotor cortex activity, decreased following a 20‐min repetitive finger typing task (Haavik Taylor & Murphy, 2007; Murphy et al., 2003). Similarly, N20 peak amplitude, which reflects activity of area 3b, was decreased after cathodal transcranial direct current stimulation (tDCS) to S1 (Dieckhofer et al., 2006), and SAI also decreased after cathodal tDCS to S1 (Kojima et al., 2015). Furthermore, B. Murphy & Dawson (2002) demonstrated that intramuscular sensation declined after a 15‐min repetitive finger typing task, and Rogalewski, Breitenstein, Nitsche, Paulus, & Knecht (2004) reported disruption of tactile perception after cathodal tDCS to the S1. Other studies have reported that P22‐N24 amplitude, which reflects S1 activity after processing by the cerebellum, increases (Andrew et al., 2015; Haavik & Murphy, 2013), and the excitability of inhibitory circuit in somatosensory cortex decreases after finger movement for 20 min (Haavik Taylor & Murphy, 2007). Therefore, we speculate that the reduction in SAI during PED has been caused by decreased inhibitory circuit excitability from S1 to M1 associated with a transient reduction in S1 activity. As we did not conduct EEG measurements, however, further studies measuring SEP waveform changes are required to confirm this hypothesis. Our results suggest that both M1 and S1 excitability reduce during PED due to decreased single MEP amplitudes and SAI after nonfatiguing finger movement.

There is a limitation in examining the change in SAI during PED. The TMS intensity used to measure SAI after the movement task was significantly higher than that before the movement task. A previous study reported that the degree of SAI measured using a high TMS intensity evoking single MEPs of 2.0 mV was smaller than that measured using a TMS intensity evoking single MEPs of 1.0 mV (Udupa et al., 2009). We cannot exclude the possibility that this higher stimulus intensity reduced SAI independently of PED.

### 4.4 Stability of SICI during PED

Our results showed that both SICI_80%RMT_ and SICI_80%AMT_ did not change during PED following nonfatiguing finger movement task. Although SICI_80%RMT_ did not change during PED (experiment 2), it is possible that this result may arise from a ceiling effect due to excessively strong suppression. Therefore, we also measured SICI_80%AMT_, which is a smaller inhibitory effect in experiment 3. However, SICI_80%AMT_ was also unchanged during PED. The previous studies reported that SICI increases during PED (Teo et al., 2012) or remains unchanged (McDonnell & Ridding, 2006). Our results are consistent with those of McDonnell and Ridding (2006) but not with those of Teo et al. (2012). The difference in results between our study and the study by Teo et al. (2012) may be due to different sample sizes (*n* = 10 in our study and *n* = 6 in the study by Teo et al., 2012) or the different motor tasks used. Additionally, PED in this study only lasted 1–2 min after the finger movement task, while PED in the previous study persisted 6–8 min after a very short 10‐s movement task (Teo et al., 2012). Therefore, PED may have been induced through different mechanisms. This difference across studies may be due to the difference of inhibitory circuits involved PED (Cholinergic inhibitory circuit in our study and GABAergic inhibitory circuits in Teo et al., 2012).

## 5 CONCLUSION

This study demonstrates that SAI decreases during PED following a nonfatiguing movement task while SICI does not change. These findings suggest that the excitability of cholinergic inhibitory circuits decrease during PED following a nonfatiguing movement task, but that the excitability of GABA_A_ circuits remains stable.

## CONFLICT OF INTERESTS

None declared.

## References

[brb3744-bib-0001] Andrew, D. , Haavik, H. , Dancey, E. , Yielder, P. , & Murphy, B. (2015). Somatosensory evoked potentials show plastic changes following a novel motor training task with the thumb. Clinical Neurophysiology, 126(3), 575–580. https://doi.org/10.1016/j.clinph.2014.05.020 24957977 10.1016/j.clinph.2014.05.020

[brb3744-bib-0002] Avanzino, L. , Tacchino, A. , Abbruzzese, G. , Quartarone, A. , Ghilardi, M. F. , Bonzano, L. , & Bove, M. (2011). Recovery of motor performance deterioration induced by a demanding finger motor task does not follow cortical excitability dynamics. Neuroscience, 174, 84–90. https://doi.org/10.1016/j.neuroscience.2010.11.008 21075172 10.1016/j.neuroscience.2010.11.008

[brb3744-bib-0003] Baumer, T. , Munchau, A. , Weiller, C. , & Liepert, J. (2002). Fatigue suppresses ipsilateral intracortical facilitation. Experimental Brain Research, 146(4), 467–473. https://doi.org/10.1007/s00221-002-1202-x 12355275 10.1007/s00221-002-1202-x

[brb3744-bib-0004] Bonato, C. , Zanette, G. , Fiaschi, A. , & Rossini, P. M. (2002). Activity‐dependent modulation of synaptic transmission in the intact human motor cortex revealed with transcranial magnetic stimulation. Cerebral Cortex, 12(10), 1057–1062. 12217969 10.1093/cercor/12.10.1057

[brb3744-bib-0005] Brasil‐Neto, J. P. , Pascual‐Leone, A. , Valls‐Sole, J. , Cammarota, A. , Cohen, L. G. , & Hallett, M. (1993). Postexercise depression of motor evoked potentials: A measure of central nervous system fatigue. Experimental Brain Research, 93(1), 181–184. 8467889 10.1007/BF00227794

[brb3744-bib-0006] Chen, R. , Corwell, B. , & Hallett, M. (1999). Modulation of motor cortex excitability by median nerve and digit stimulation. Experimental Brain Research, 129(1), 77–86. 10550505 10.1007/s002210050938

[brb3744-bib-0007] Crupi, D. , Cruciata, G. , Moisello, C. , Green, P. A. , Naro, A. , Ricciardi, L. , & Ghilardi, M. F. (2013). Protracted exercise without overt neuromuscular fatigue influences cortical excitability. Journal of Motor Behavior, 45(2), 127–138. https://doi.org/10.1080/00222895.2012.760514 23488595 10.1080/00222895.2012.760514

[brb3744-bib-0008] Cunningham, D. A. , Janini, D. , Wyant, A. , Bonnett, C. , Varnerin, N. , Sankarasubramanian, V. , & Plow, E. B. (2016). Post‐exercise depression following submaximal and maximal isometric voluntary contraction. Neuroscience, 326, 95–104. https://doi.org/10.1016/j.neuroscience.2016.03.060 27058145 10.1016/j.neuroscience.2016.03.060 PMC7062044

[brb3744-bib-0009] Di Lazzaro, V. , Oliviero, A. , Profice, P. , Pennisi, M. A. , Di Giovanni, S. , Zito, G. , & Rothwell, J. C. (2000). Muscarinic receptor blockade has differential effects on the excitability of intracortical circuits in the human motor cortex. Experimental Brain Research, 135(4), 455–461. 11156309 10.1007/s002210000543

[brb3744-bib-0010] Di Lazzaro, V. , Oliviero, A. , Saturno, E. , Dileone, M. , Pilato, F. , Nardone, R. , & Tonali, P. (2005a). Effects of lorazepam on short latency afferent inhibition and short latency intracortical inhibition in humans. Journal of Physiology, 564(Pt 2), 661–668. https://doi.org/10.1113/jphysiol.2004.061747 15718269 10.1113/jphysiol.2004.061747 PMC1464438

[brb3744-bib-0011] Di Lazzaro, V. , Pilato, F. , Dileone, M. , Tonali, P. A. , & Ziemann, U. (2005b). Dissociated effects of diazepam and lorazepam on short‐latency afferent inhibition. Journal of Physiology, 569(Pt 1), 315–323. https://doi.org/10.1113/jphysiol.2005.092155 16141274 10.1113/jphysiol.2005.092155 PMC1464195

[brb3744-bib-0012] Dieckhofer, A. , Waberski, T. D. , Nitsche, M. , Paulus, W. , Buchner, H. , & Gobbele, R. (2006). Transcranial direct current stimulation applied over the somatosensory cortex ‐ differential effect on low and high frequency SEPs. Clinical Neurophysiology, 117(10), 2221–2227. https://doi.org/10.1016/j.clinph.2006.07.136 16931142 10.1016/j.clinph.2006.07.136

[brb3744-bib-0013] Fischer, M. , & Orth, M. (2011). Short‐latency sensory afferent inhibition: Conditioning stimulus intensity, recording site, and effects of 1 Hz repetitive TMS. Brain Stimulation, 4(4), 202–209. https://doi.org/10.1016/j.brs.2010.10.005 22032735 10.1016/j.brs.2010.10.005

[brb3744-bib-0014] Gandevia, S. C. , Petersen, N. , Butler, J. E. , & Taylor, J. L. (1999). Impaired response of human motoneurones to corticospinal stimulation after voluntary exercise. Journal of Physiology, 521(Pt 3), 749–759. 10601504 10.1111/j.1469-7793.1999.00749.x PMC2269689

[brb3744-bib-0015] Haavik, H. , & Murphy, B. A. (2013). Selective changes in cerebellar‐cortical processing following motor training. Experimental Brain Research, 231(4), 397–403. https://doi.org/10.1007/s00221-013-3704-0 24065291 10.1007/s00221-013-3704-0

[brb3744-bib-0016] Haavik Taylor, H. , & Murphy, B. A. (2007). Altered cortical integration of dual somatosensory input following the cessation of a 20 min period of repetitive muscle activity. Experimental Brain Research, 178(4), 488–498. https://doi.org/10.1007/s00221-006-0755-5 17136532 10.1007/s00221-006-0755-5

[brb3744-bib-0017] Khan, S. I. , Giesebrecht, S. , Gandevia, S. C. , & Taylor, J. L. (2012). Activity‐dependent depression of the recurrent discharge of human motoneurones after maximal voluntary contractions. Journal of Physiology, 590(19), 4957–4969. https://doi.org/10.1113/jphysiol.2012.235697 22907051 10.1113/jphysiol.2012.235697 PMC3487048

[brb3744-bib-0018] Kluger, B. M. , Palmer, C. , Shattuck, J. T. , & Triggs, W. J. (2012). Motor evoked potential depression following repetitive central motor initiation. Experimental Brain Research, 216(4), 585–590. https://doi.org/10.1007/s00221-011-2962-y 22130780 10.1007/s00221-011-2962-y PMC12930409

[brb3744-bib-0019] Kojima, S. , Onishi, H. , Miyaguchi, S. , Kotan, S. , Sugawara, K. , Kirimoto, H. , & Tamaki, H. (2015). Effects of cathodal transcranial direct current stimulation to primary somatosensory cortex on short‐latency afferent inhibition. NeuroReport, 26(11), 634–637. https://doi.org/10.1097/wnr.0000000000000402 26103117 10.1097/WNR.0000000000000402

[brb3744-bib-0020] Kujirai, T. , Caramia, M. D. , Rothwell, J. C. , Day, B. L. , Thompson, P. D. , Ferbert, A. , & Marsden, C. D. (1993). Corticocortical inhibition in human motor cortex. Journal of Physiology, 471, 501–519. 8120818 10.1113/jphysiol.1993.sp019912 PMC1143973

[brb3744-bib-0021] Liepert, J. , Kotterba, S. , Tegenthoff, M. , & Malin, J. P. (1996). Central fatigue assessed by transcranial magnetic stimulation. Muscle and Nerve, 19(11), 1429–1434. https://doi.org/10.1002/(SICI)1097-4598(199611)19:11<1429:AID-MUS7>3.0.CO;2-E 8874400 10.1002/(SICI)1097-4598(199611)19:11<1429::AID-MUS7>3.0.CO;2-E

[brb3744-bib-0022] Maruyama, A. , Matsunaga, K. , Tanaka, N. , & Rothwell, J. C. (2006). Muscle fatigue decreases short‐interval intracortical inhibition after exhaustive intermittent tasks. Clinical Neurophysiology, 117(4), 864–870. https://doi.org/10.1016/j.clinph.2005.12.019 16495147 10.1016/j.clinph.2005.12.019

[brb3744-bib-0023] McDonnell, M. N. , & Ridding, M. C. (2006). Transient motor evoked potential suppression following a complex sensorimotor task. Clinical Neurophysiology, 117(6), 1266–1272. https://doi.org/10.1016/j.clinph.2006.02.008 16600678 10.1016/j.clinph.2006.02.008

[brb3744-bib-0024] Miyaguchi, S. , Kojima, S. , Kirimoto, H. , Tamaki, H. , & Onishi, H. (2016). Do differences in levels, types, and duration of muscle contraction have an effect on the degree of post‐exercise depression? Frontiers in Human Neuroscience, 10, 159 https://doi.org/10.3389/fnhum.2016.00159 27199696 10.3389/fnhum.2016.00159 PMC4850151

[brb3744-bib-0025] Miyaguchi, S. , Onishi, H. , Kojima, S. , Sugawara, K. , Tsubaki, A. , Kirimoto, H. , & Yamamoto, N. (2013). Corticomotor excitability induced by anodal transcranial direct current stimulation with and without non‐exhaustive movement. Brain Research, 1529, 83–91. https://doi.org/10.1016/j.brainres.2013.07.026 23891715 10.1016/j.brainres.2013.07.026

[brb3744-bib-0026] Murphy, B. A. , Haavik Taylor, H. , Wilson, S. A. , Oliphant, G. , & Mathers, K. M. (2003). Rapid reversible changes to multiple levels of the human somatosensory system following the cessation of repetitive contractions: A somatosensory evoked potential study. Clinical Neurophysiology, 114(8), 1531–1537. 12888037 10.1016/s1388-2457(03)00127-5

[brb3744-bib-0027] Ni, Z. , Charab, S. , Gunraj, C. , Nelson, A. J. , Udupa, K. , Yeh, I. J. , & Chen, R. (2011). Transcranial magnetic stimulation in different current directions activates separate cortical circuits. Journal of Neurophysiology, 105(2), 749–756. https://doi.org/10.1152/jn.00640.2010 21148098 10.1152/jn.00640.2010

[brb3744-bib-0028] Petersen, N. T. , Taylor, J. L. , Butler, J. E. , & Gandevia, S. C. (2003). Depression of activity in the corticospinal pathway during human motor behavior after strong voluntary contractions. Journal of Neuroscience, 23(22), 7974–7980. 12954858 10.1523/JNEUROSCI.23-22-07974.2003 PMC6740505

[brb3744-bib-0029] Roshan, L. , Paradiso, G. O. , & Chen, R. (2003). Two phases of short‐interval intracortical inhibition. Experimental Brain Research, 151(3), 330–337. https://doi.org/10.1007/s00221-003-1502-9 12802553 10.1007/s00221-003-1502-9

[brb3744-bib-0030] Samii, A. , Wassermann, E. M. , Ikoma, K. , Mercuri, B. , & Hallett, M. (1996). Characterization of postexercise facilitation and depression of motor evoked potentials to transcranial magnetic stimulation. Neurology, 46(5), 1376–1382. 8628485 10.1212/wnl.46.5.1376

[brb3744-bib-0031] Sasaki, R. , Miyaguchi, S. , Kotan, S. , Kojima, S. , Kirimoto, H. , & Onishi, H. (2016). Modulation of cortical inhibitory circuits after cathodal transcranial direct current stimulation over the primary motor cortex. Frontiers in Human Neuroscience, 10, 30 https://doi.org/10.3389/fnhum.2016.00030 26869909 10.3389/fnhum.2016.00030 PMC4740366

[brb3744-bib-0032] Teo, W. P. , Rodrigues, J. P. , Mastaglia, F. L. , & Thickbroom, G. W. (2012). Post‐exercise depression in corticomotor excitability after dynamic movement: A general property of fatiguing and non‐fatiguing exercise. Experimental Brain Research, 216(1), 41–49. https://doi.org/10.1007/s00221-011-2906-6 22038716 10.1007/s00221-011-2906-6

[brb3744-bib-0033] Teo, J. T. , Terranova, C. , Swayne, O. , Greenwood, R. J. , & Rothwell, J. C. (2009). Differing effects of intracortical circuits on plasticity. Experimental Brain Research, 193(4), 555–563. https://doi.org/10.1007/s00221-008-1658-4 19048237 10.1007/s00221-008-1658-4 PMC3019102

[brb3744-bib-0034] Tokimura, H. , Di Lazzaro, V. , Tokimura, Y. , Oliviero, A. , Profice, P. , Insola, A. , & Rothwell, J. C. (2000). Short latency inhibition of human hand motor cortex by somatosensory input from the hand. Journal of Physiology, 523(Pt 2), 503–513. 10699092 10.1111/j.1469-7793.2000.t01-1-00503.x PMC2269813

[brb3744-bib-0035] Tsang, P. , Jacobs, M. F. , Lee, K. G. , Asmussen, M. J. , Zapallow, C. M. , & Nelson, A. J. (2014). Continuous theta‐burst stimulation over primary somatosensory cortex modulates short‐latency afferent inhibition. Clinical Neurophysiology, 125(11), 2253–2259. https://doi.org/10.1016/j.clinph.2014.02.026 24775920 10.1016/j.clinph.2014.02.026

[brb3744-bib-0036] Udupa, K. , Ni, Z. , Gunraj, C. , & Chen, R. (2009). Interactions between short latency afferent inhibition and long interval intracortical inhibition. Experimental Brain Research, 199(2), 177–183. https://doi.org/10.1007/s00221-009-1997-9 19730839 10.1007/s00221-009-1997-9

[brb3744-bib-0037] Zanette, G. , Bonato, C. , Polo, A. , Tinazzi, M. , Manganotti, P. , & Fiaschi, A. (1995). Long‐lasting depression of motor‐evoked potentials to transcranial magnetic stimulation following exercise. Experimental Brain Research, 107(1), 80–86. 8751065 10.1007/BF00228019

[brb3744-bib-0038] Ziemann, U. , Netz, J. , Szelenyi, A. , & Homberg, V. (1993). Spinal and supraspinal mechanisms contribute to the silent period in the contracting soleus muscle after transcranial magnetic stimulation of human motor cortex. Neuroscience Letters, 156(1–2), 167–171. 8414181 10.1016/0304-3940(93)90464-v

[brb3744-bib-0039] Ziemann, U. , Rothwell, J. C. , & Ridding, M. C. (1996). Interaction between intracortical inhibition and facilitation in human motor cortex. Journal of Physiology, 496(Pt 3), 873–881. 8930851 10.1113/jphysiol.1996.sp021734 PMC1160871

